# Behavioral and Health Correlates of Resting-State Metastability in the Human Connectome Project

**DOI:** 10.1007/s10548-018-0672-5

**Published:** 2018-08-22

**Authors:** Won Hee Lee, Dominik Andreas Moser, Alex Ing, Gaelle Eve Doucet, Sophia Frangou

**Affiliations:** 1 Department of Psychiatry, Icahn School of Medicine at Mount Sinai, 1425 Madison Avenue, New York, NY 10029 USA; 2 Institute of Psychiatry, Psychology and Neuroscience, King’s College London, London, UK

**Keywords:** Resting-state fMRI, Metastability, Human Connectome Project, Canonical correlation analysis, Body mass index

## Abstract

Metastability is currently considered a fundamental property of the functional configuration of brain networks. The present study sought to generate a normative reference framework for the metastability of the major resting-state networks (RSNs) (resting-state metastability dataset) and discover their association with demographic, behavioral, physical and cognitive features (non-imaging dataset) from 818 participants of the Human Connectome Project. Using sparse canonical correlation analysis, we found that the metastability and non-imaging datasets showed significant but modest interdependency. Notable associations between the metastability variate and the non-imaging features were observed for higher-order cognitive ability and indicators of physical well-being. The intra-class correlation coefficient between the sibling pairs in the sample was very low which argues against a significant familial influence on RSN metastability.

**Electronic supplementary material:**

The online version of this article (10.1007/s10548-018-0672-5) contains supplementary material, which is available to authorized users.

## Introduction

Recent conceptual and methodological advances have shifted the focus of research to the dynamic properties of brain networks (Deco et al. 2015; Kelso 2012) that reflect the coupling and de-coupling of brain regions over time. The central assumption is that mental operations are the emerging properties of neural communication, which is predicated on the coherent and flexible oscillatory activity between neural ensembles (Harris and Gordon 2015; Tognoli and Kelso 2014). The concept of metastability has been introduced to describe the repertoire of functional configurations arising from temporal variations in this oscillatory activity of a network’s constituent regions (Deco and Kringelbach 2016). It is though that optimal brain function occurs within a range of metastability that reflects the balance between synchronization and adaptive reconfiguration of the functional connections between a network’s constituent regions (Cabral et al. 2011; Deco and Kringelbach 2016; Hellyer et al. 2014; Senden et al. 2017). Thus, examination of metastability may provide novel insights into the pathophysiology of neurological (Alstott et al. 2009; Hellyer et al. 2015; Honey and Sporns 2008) and psychiatric disorders (Cabral et al. 2013; Cordova-Palomera et al. 2017).

A range of computational models has been used to capture the metastability of human brain networks (Cabral et al. 2011, 2017; Deco et al. 2017; Lee and Frangou 2017). Here, we used functional magnetic resonance imaging (fMRI) blood-oxygen-level dependent (BOLD) signal from a network’s constituent regions to compute the metastability of that network as the temporal variation of phase synchronization based on the Kuramoto order parameter (Wildie and Shanahan 2012). We were particularly interested in the metastability of the resting-state networks (RSNs) that define the intrinsic functional architecture of the brain (Biswal et al. 1995). Specifically, we focused on the default mode (DMN), the central executive (CEN), the salience (SAL), the dorsal attention (DAN), the language (LAN), the auditory (AN), the visual (VN), and the sensorimotor (SMN) networks (Damoiseaux et al. 2006; Power et al. 2011; Smith et al. 2009). The spatial patterns of RSNs are generally consistent across healthy individuals (Damoiseaux et al. 2006; Shehzad et al. 2009) and are under partial genetic control (Fornito et al. 2011; Glahn et al. 2010). RSNs are aligned with functional networks derived from task-based fMRI studies; specifically lower-order networks (e.g., AN, VN) are primarily involved in circumscribed and specialized functions while higher-order networks (e.g., CEN, DMN) are involved in diverse and complex mental operations (Smith et al. 2009). Further, Smith and colleagues (Smith et al. 2015) have demonstrated that resting-state brain connectivity has wide functional implications as it is linked to human traits along a positive–negative axis: features or attributes (e.g., IQ, physical endurance) considered correlated positively with resting-state connectivity while the reverse was the case for “negative” characteristics (e.g., substance use).

We are not aware of previous studies to have examined the normative range of RSN metastability and to have sought to integrate these measures with human physical and behavioral characteristics. Normative data are typically obtained from a representative sample from the wider population and can be used to establish the baseline distribution for a measurement. To address the knowledge gap, we used the rich dataset of the Human Connectome Project (HCP; http://www.humanconnectome.org). We analyzed resting-state fMRI data from 818 HCP participants to define the normative range of metastability of the DMN, CEN, SAL, DAN, LAN, AN, VN and SMN, and we used sparse canonical correlation analyses to discover patterns of association between the metastability of different RSNs and demographic, behavioral, physical and cognitive features.

## Materials and Methods

### Participants

The study sample consisted of 818 healthy HCP participants (459 women) with a mean age of 29 years (range 22–37 years). The majority of the HCP participants were siblings while 370 individuals were unrelated.

### HCP Neuroimaging Data Acquisition and Quality Assurance

We used publicly available resting-state fMRI data acquired on a Siemens Skyra 3 T scanner as part of the HCP (Glasser et al. 2013). All data were de-identified prior to release as described by Van Essen and Barch (Van Essen and Barch 2015). Data preprocessing and quality control (including head motion) were implemented through the HCP pipeline, as detailed by Glasser et al. (2013), using tools from FSL (Jenkinson et al. 2012), FreeSurfer (Fischl et al. 1999) and the HCP workbench (Marcus et al. 2013). An additional preprocessing step was applied to minimize head motion by removing structured artifacts using an automatic denoising approach based on independent component analysis (ICA) followed by FMRIB’s ICA-based X-noiseifier (Griffanti et al. 2014; Salimi-Khorshidi et al. 2014).

### Computation of Metastability of RSNs

To enhance reproducibility, RSNs were defined in each participant using the functional templates available through the Functional Imaging in Neuropsychiatric Disorders Lab at Stanford University, USA (https://findlab.stanford.edu/functional_ROIs.html) (Supplementary Figure S1 and Table S1) (Shirer et al. 2012). We specifically examined the dorsal attention network (DAN), the central executive network (CEN), the salience network (SAL), the somatosensory network (SMN), the visual network (VN), the auditory network (AN), and the language network (LAN). We considered the default mode network (DMN) in terms of its sub-divisions into the dorsal DMN (dDMN), the ventral DMN (vDMN) and the precuneus network (PN) because prior evidence indicates that each DMN sub-division may support different cognitive processes (Andrews-Hanna et al. 2010) and may have a different functional impact on whole brain organization (Doucet et al. 2011). In each participant, we calculated the average time-series of all the voxels in each region of each RSN, and then applied the bandpass filtering (0.01–0.1 Hz) to isolate low-frequency resting-state blood oxygen-level dependent (BOLD) signal fluctuations (Cordes et al. 2001). The Hilbert transform was applied to the bandpass-filtered fMRI signals to compute the associated analytical signals. The analytic signal represents a narrowband signal, $s\left(t\right)$, in the time domain as a rotating vector with an instantaneous phase, $\phi \left(t\right)$, and an instantaneous amplitude, $A\left(t\right)$, i.e., $s\left(t\right)=A\left(t\right)\text{cos}\left(\phi \left(t\right)\right)$. The phase and the amplitude are given by the argument and the modulus, respectively, of the complex signal $z\left(t\right)$, given by $z\left(t\right)=s\left(t\right)+i.\text{H}\left[s\left(t\right)\right]$, where $i$ is the imaginary unit and $\text{H}\left[s\left(t\right)\right]$ is the Hilbert transform of $s\left(t\right)$ (Glerean et al. 2012). To evaluate the dynamic properties of each RSN, we computed the Kuramoto order parameter $R\left(t\right)$, defined as

$$R\left( t \right)=\frac{1}{N}\left| {~\mathop \sum \limits_{{n=1}}^{N} {e^{i{\varphi _n}(t)}}~} \right|$$

where *N* is the total number of regions within each RSN and ${\phi }_{n}\left(t\right)$ is the instantaneous phase of the BOLD signal at region *n* of each RSN. For each RSN, metastability was defined as the standard deviation of the Kuramoto order parameter $R\left(t\right)$ over time (Cabral et al. 2011; Lee et al. 2017; Lee and Frangou 2017; Shanahan 2010).

### Datasets Entered in Sparse Canonical Correlation Analyses

We considered the covariation patterns between two datasets: the resting-state metastability dataset and the non-imaging dataset. The former comprised the metastability measures of the RSNs defined in the previous sections. The non-imaging dataset comprised 112 variables corresponding to demographic characteristics, cognitive task performance, mental health and personality, physical health and lifestyle choices detailed in Supplementary Table S2. These are a subset of the phenotypic variables provided by the HCP Dataset. In constructing the non-imaging dataset used here, (a) we selected age-adjusted cognitive test scores; (b) we excluded co-linear continuous variables (r > 0.9) variables; (d) for psychometric tests with multiple correlated outcome variables, we selected those most commonly reported in the literature; (d) we excluded categorical variables for which more than 90% of the sample endorsed the same outcome.

### Sparse Canonical Correlation Analyses (sCCA)

We used sparse Canonical Correlation Analysis (sCCA) to test the association between the resting-state metastability and the non-imaging datasets. We chose a sparse multivariate approach because it does not require data reduction, regardless of the number of subjects and variables. sCCA can be used in smaller samples (that are more typical of neuroimaging studies) and is less susceptible to overfitting than classical CCA. For the sCCA, (a) we computed the sparse parameters for a range of candidate values from 0.1 × √p (high sparsity) to 1 × √p (low sparsity) at increments of 0.1, where p is the number of features in each data set, and fitted the resulting models; (b) we selected the optimal sparse criteria combination based on the parameters that corresponded to the values that maximized the sCCA correlation value. These optimal criteria were 0.4 × √p for the non-imaging dataset and 0.8 × √p for the imaging dataset; (c) we determined the optimal sCCA model and established its significance at p value < 0.05 using permutations (n = 10,000). The p value was defined as the number of permutations that resulted in a higher correlation than the original data divided by the total number of permutations. Thus, the p value was explicitly corrected for multiple testing as it was compared against the null distribution of maximal correlation values across sCCAs estimated for each combination of sparsity parameters. When the overall sCCA was significant, we calculated the correlations between the individual variables and the variate of the opposite dataset (i.e. the output of the sCCA for that dataset). We also extracted the weights that each variable contributed towards the canonical correlation with the opposite variate.

### Reliability and Robustness of the sCCA analyses

We considered sex, age, education, date of the acquisition and mean head motion as potential a priori confounders. Prior to the sCCA, we performed univariate tests between each confounder and the metastability of the resting state networks. Potential confounding variables that showed at least one significant univariate association at p < 0.05 uncorrected were included in the non-imaging dataset. On this basis, education, head motion, age and date of acquisition were retained for sCCA analyses.

To confirm the robustness of the sCCA results we created training datasets by randomly resampling half of the sample 10,000 times and repeating the sCCAs on these sets. These sCCAs yielded similar results as those obtained from the original full dataset: the mean correlation of the sCCA models resulting from the 10,000 random resampled datasets was r = 0.28 (standard deviation = 0.03) which indicated that the size of the sCCA correlation between the metastability and non-imaging variates was stable and that overfitting was minimal. We also performed the leave-one-out analyses that reinforced the stability of the sCCA results because the weights of each leave-one-out test correlated above 0.98 with the sCCA weights in the full dataset.

### Intraclass Correlation Coefficient Analyses

We assessed familial similarity in metastability in sibling pairs using the intraclass correlation coefficient (ICC) (McGraw and Wong 1996; Shrout and Fleiss 1979). No further heritability analyses were performed due to the lack of familial associations.

### Supplemental Analyses

We conducted the following supplemental analyses: (i) we provided the centile values of the metastability of each RSN as shown in Supplementary Table S3 and Supplementary Figure S2; (ii) we further characterized the correlation matrix of our datasets by computing the univariate Pearson’s correlation between each of the non-imaging variables and each of the resting-state metastability variables as shown in Supplementary Figure S4; (iii) although our focus is on metastability, we examined resting-state network synchrony using the same approach and we present the results in the Supplementary Material; (iv) we conducted a further sCCA analysis following the same procedures described above; this supplemental sCCA involved the non-imaging dataset and a new dataset that comprised the pairwise differences in RSN metastability. These results of the supplemental analyses are presented in the Supplementary Material.

## Results

### Metastability of the Major RSNs

**Fig. 1:**
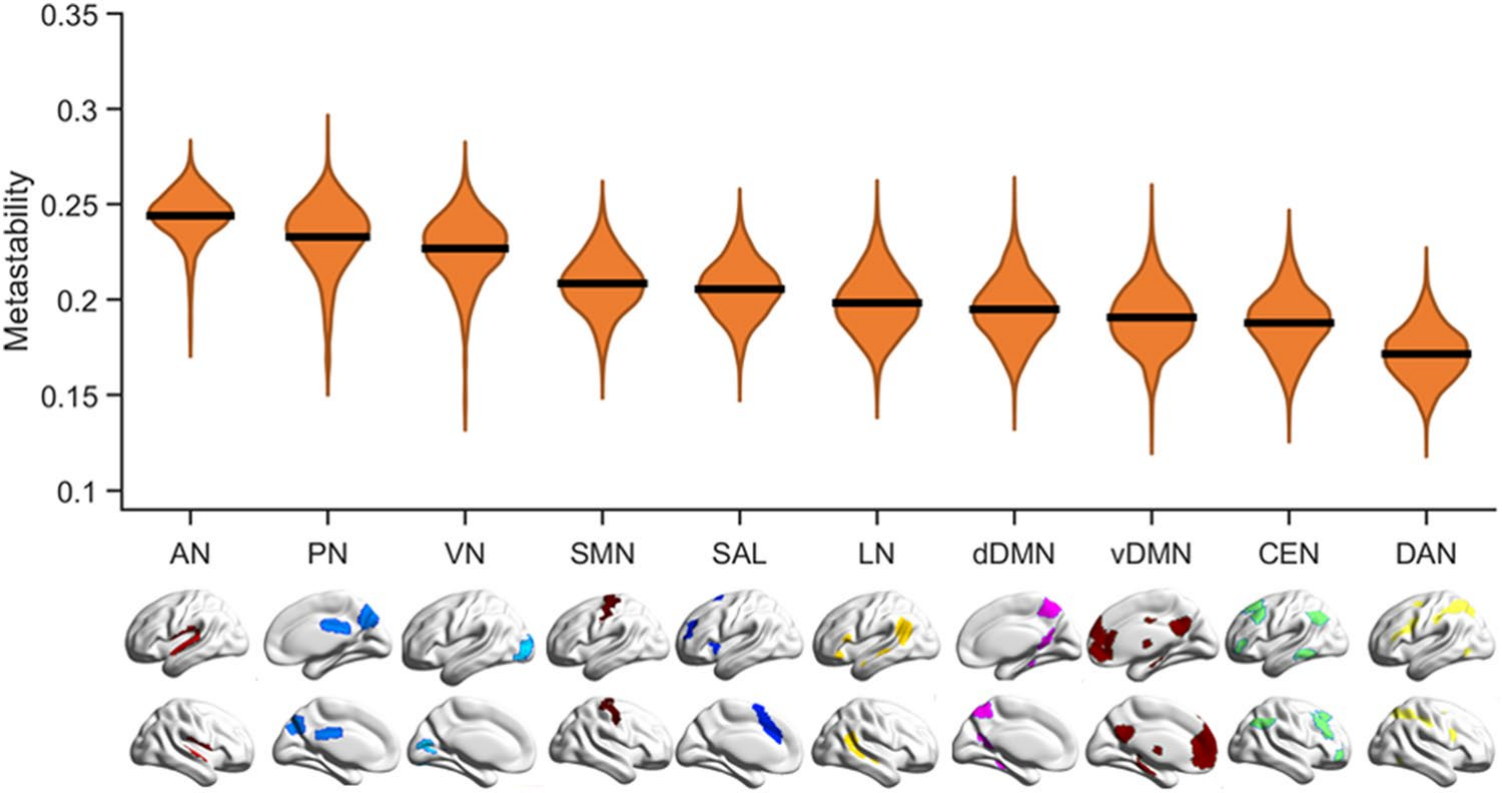
Violin plots of the metastability distribution for each resting-state network (RSN) across the 818 participants of the Human Connectome Project. The solid black lines depict mean values across participants. *AN* auditory network, *PN* precuneus network, *VN* visual network, *SMN* sensorimotor network, *SAL* salience network, *LN* language network, *dDMN* dorsal default mode network, *vDMN* ventral default mode network, *CEN* central executive network, *DAN* dorsal attention network

Figure 1 shows violin plots of the distribution of metastability measures across participants for each RSN. Analysis of variance (F_9_ = 1727.13, p < 10^−15^) and Bonferroni corrected post-hoc pairwise comparisons (n = 45) showed that metastability differed significantly between networks (details in Supplementary Table S4). The Bonferroni adjusted p value was set at 0.001.

### Covariation Patterns Between the Metastability and Non-imaging Datasets

**Fig. 2:**
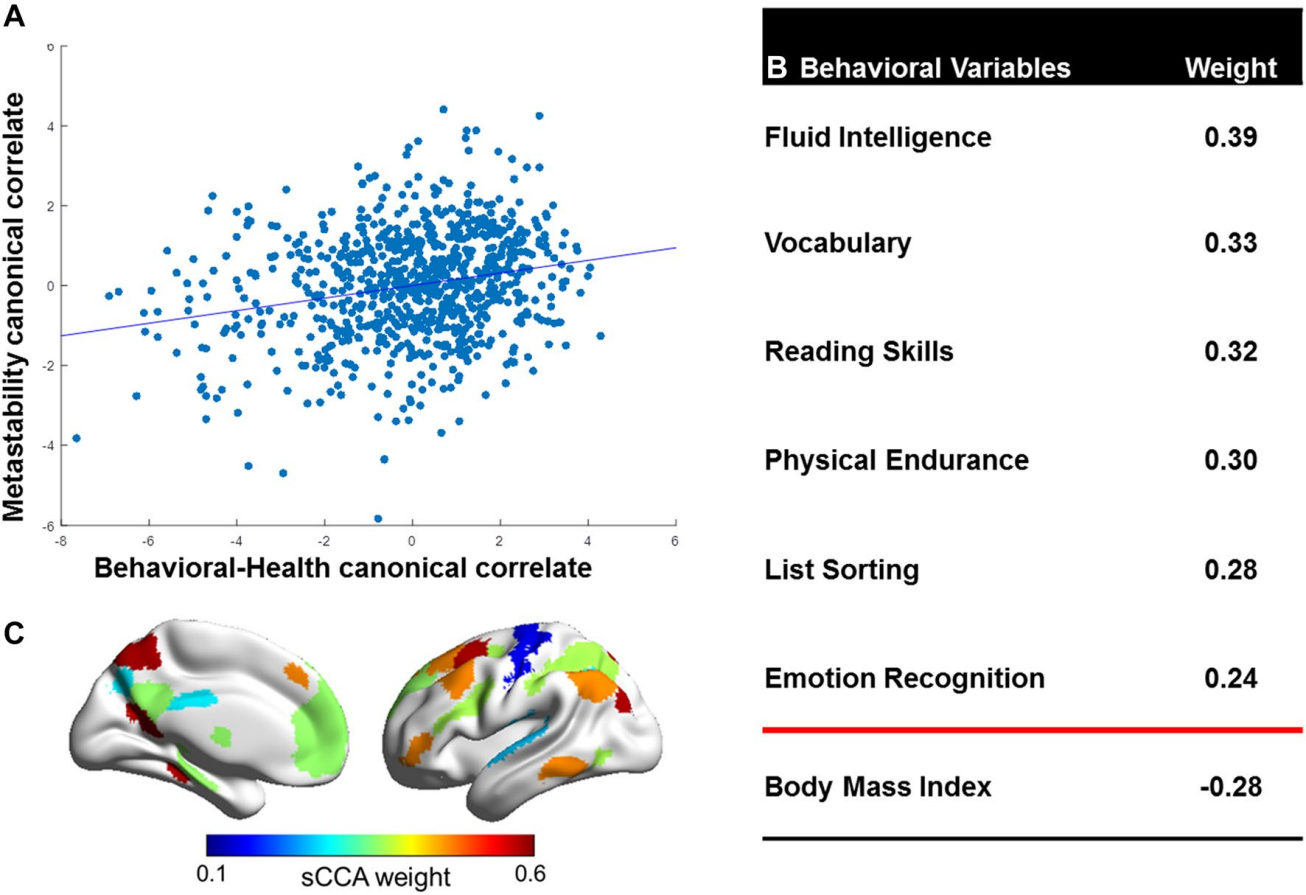
**a** Association between resting-state network (RSN) metastability and non-imaging variates following sparse canonical correlation analysis (r = 0.23, p = 0.03). **b** Non-imaging variables with the highest weights of association with the RSN-metastability variate (details in Supplementary Table S5). **c** RSNs with the highest weights of association with the non-imaging variate (details in Supplementary Table S6 and Supplemental Figure S6)

For the first mode, the sCCA between the two datasets was modest but significant (r = 0.23, p = 0.03, Fig. 2a) and robust (Supplementary Figure S5). We report on the first mode only which explained most of covariation between datasets since all other modes were not significant (second: r = 0.19, p = 0.27; third: r = 0.21, p = 0.30).

The non-imaging variables which had the highest covariation with the metastability variate were fluid intelligence (weight = 0.39), reading skills (weight = 0.33), emotion recognition (weight = 0.24), physical endurance (weight = 0.28), body mass index (BMI, weight = − 0.28) and amount of sleep (weight = 0.11) as shown in Fig. 2b and Supplementary Table S5. Metastability variables that showed the highest covariation with the non-imaging variate involved the three DMN sub-divisions (vDMN: weight = 0.58, dDMN: weight = 0.37, PN: weight = 0.28), the CEN (weight = 0.47) and the DAN (weight = 0.37) as shown in Fig. 2c, Supplementary Table S6 and Supplementary Figure S6. None of the potential confounding variables (which included head motion) correlated with the metastability variate (Supplementary Tables S7 and S8).

### Intraclass Correlation Coefficient Analyses

Across all RSNs, the ICC of metastability between pairs of siblings was small (ICC < 0.11) and not statistically significant following correction for multiple testing. Therefore, no further analyses of heritability were performed.

## Discussion

We used data from 818 HCP participants to estimate metastability measures of the major RSNs and determined their association with demographic, behavioral, physical and cognitive features. This study benefits from the unique dataset of the HCP which is substantial in terms of its size and richness of the phenotypic measures. The metastability of the RSNs presented can be considered representative for samples of young adults and can be used to assess the generalizability of future studies.

In general, higher metastability was noted in lower-order RSNs, such as the AN and VN, that are involved in specialized and mostly externally-driven functions. The higher levels of metastability of these networks may reflect greater capacity in altering their functional configuration in order to respond to diverse and rapidly changing external inputs (Power et al. 2011; Smith et al. 2009). On the other hand, higher-order networks such as the DMN and CEN, implicated mostly in internal and goal-directed processing (Greicius et al. 2003; Raichle et al. 2001), had lower metastability. These results indicate that higher-order networks tend to maintain their functional configurations over longer time scale consistent with their primary function in supporting sustained mental operations. Interestingly, the SAL network exhibited intermediate levels of metastability, aligned with its proposed central role in switching between higher and lower-order networks (Menon and Uddin 2010; Seeley et al. 2007).

We found that the association between RSN metastability and human traits was robust but modest. This modest degree of covariation may be characteristic of the dynamic architecture of the brain at rest. It is likely to reflect what Deco et al. (2011) described as “a constant inner state of exploration,” in which the brain at rest remains in a state of predictive readiness but does not commit to specific network configurations until required. The small ICC of metastability between pairs of siblings for all RSNs suggests that familial influences on metastability are minimal. This contrasts with the significant genetic influence on conventional resting-state functional connectivity, particularly of the DMN (Glahn et al. 2010). This finding further supports the notion that dynamic brain states at rest are less constrained by either genetic or other non-imaging factors and are more likely to be influenced by the immediate demands on brain function.

Non-imaging variables that positively associated with the metastability variate corresponded to positive cognitive attributes (Fig. 2b). This pattern is consistent with previous reports using the HCP dataset where positive cognitive attributes showed the highest covariation with the functional connectome (Moser et al. 2017; Smith et al. 2015). These findings underscore the relevance of both functional and dynamic connectivity for higher-order cognitive abilities. In parallel, emerging evidence from clinical populations, such as patients with Alzheimer’s disease (Cordova-Palomera et al. 2017) and traumatic brain injury (Hellyer et al. 2015), shows that decline in cognitive capacity is associated with reduction in resting-state metastability. It would therefore appear that diverse pathogenetic mechanisms that affect the brain may also act to reduce the dynamic repertoire of its resting-state networks. Physical endurance and amount of sleep were positively associated with the metastability variate while the opposite was the case for BMI. These findings link the dynamic properties of resting-state networks to indicators of physical well-being. Similar associations have been reported in terms of conventional resting-state connectivity (Miller et al. 2016; Smith et al. 2015) and emphasize the importance of considering physical traits when interpreting variation in brain metrics.

This study has several limitations. This work focused solely on the RSN metastability, leaving aside other properties of the RSNs or computational modeling. We chose to focus on metastability given the wide use of this measure in neuropsychiatric research while future studies could widen the scope of examination of the dynamic properties of the RSNs beyond that considered here. Similarly, the non-imaging dataset did not include all theoretically possible features; it does encompass however multiple human traits that are most likely to be relevant to resting-state brain organization. We used templates to define the RSNs to enhance the reproducibility of the study findings in preference to other methods for partitioning the resting-state connectome. The most widely used alternative is to use independent component analyses to identify RSN. This approach may be more sensitive to individual-level variability while functionally defined templates such as the one employed here have the advantage of greater cogency (Laird et al. 2011; Power et al. 2011; Smith et al. 2009).

## Conclusions

This is the first study to provide a normative framework for resting-state network metastability, and demonstrate modest but meaningful associations of this property to higher-order cognitive abilities and physical indicators of well-being.

## Electronic supplementary material

Below is the link to the electronic supplementary material.

Supplementary material 1 (DOCX 1079 KB)
