# Supplementary material for: Behavioral and Health Correlates of Resting-State Metastability in the Human Connectome Project
Source: Brain Topogr. 2018 Aug 22;32(1):80–6. doi: 10.1007/s10548-018-0672-5 (PMC6326990; doi:10.1007/s10548-018-0672-5)
Supplement: Supplementary file 1 — Supplementary material 1 (DOCX 1079 KB) [file 10548_2018_672_MOESM1_ESM.docx]

**Supplementary Material**

**Behavioral and health correlates of resting-state metastability in the Human Connectome Project**

Won Hee Lee, Dominik Andreas Moser, Alex Ing, Gaelle Eve Doucet, Sophia Frangou

**Contents:**

1. **Description of imaging and non-imaging parameters**

Figure S1. Spatial maps of the resting-state networks

Table S1. Regions comprising the resting-state networks

Table S2. Definition of non-imaging variables

1. **Properties of resting-state network metastability**

Table S3. Percentile values of the metastability of each resting-state network

Figure S2. Percentile curves of the metastability of each resting-state network

Figure S3. Histograms of the metastability of each resting-state network

Table S4. Bonferroni corrected pairwise comparisons of the metastability of the resting-state networks

Figure S4. Univariate Pearson’s correlations between the variables of the resting-state metastability dataset and the non-imaging dataset

Table S5. Canonical correlation coefficients and weights between the metastability variate and the non-imaging variables

Figure S5. Distribution of the sparse canonical correlation coefficients based on 10,000 randomly resampled subsets of half the sample (n=409)

Table S6. Canonical correlation coefficients and weights between the non-imaging variate and the metastability variables

Figure S6. Sparse canonical correlation analysis (sCCA) weights of the resting-state networks with the non-imaging variate

1. **Potential Confounders**

Table S7. Association of the metastability variate with potentially confounding variables in the non-imaging dataset

Table S8. Univariate correlations between resting-state metastability and head motion

1. **Network Synchrony**

Figure S7. Violin plots of the distribution of synchrony for each resting-state network (RSN)

Table S9. Univariate correlations between synchrony and metastability for each resting-state network

**A. Description of imaging and non-imaging parameters**


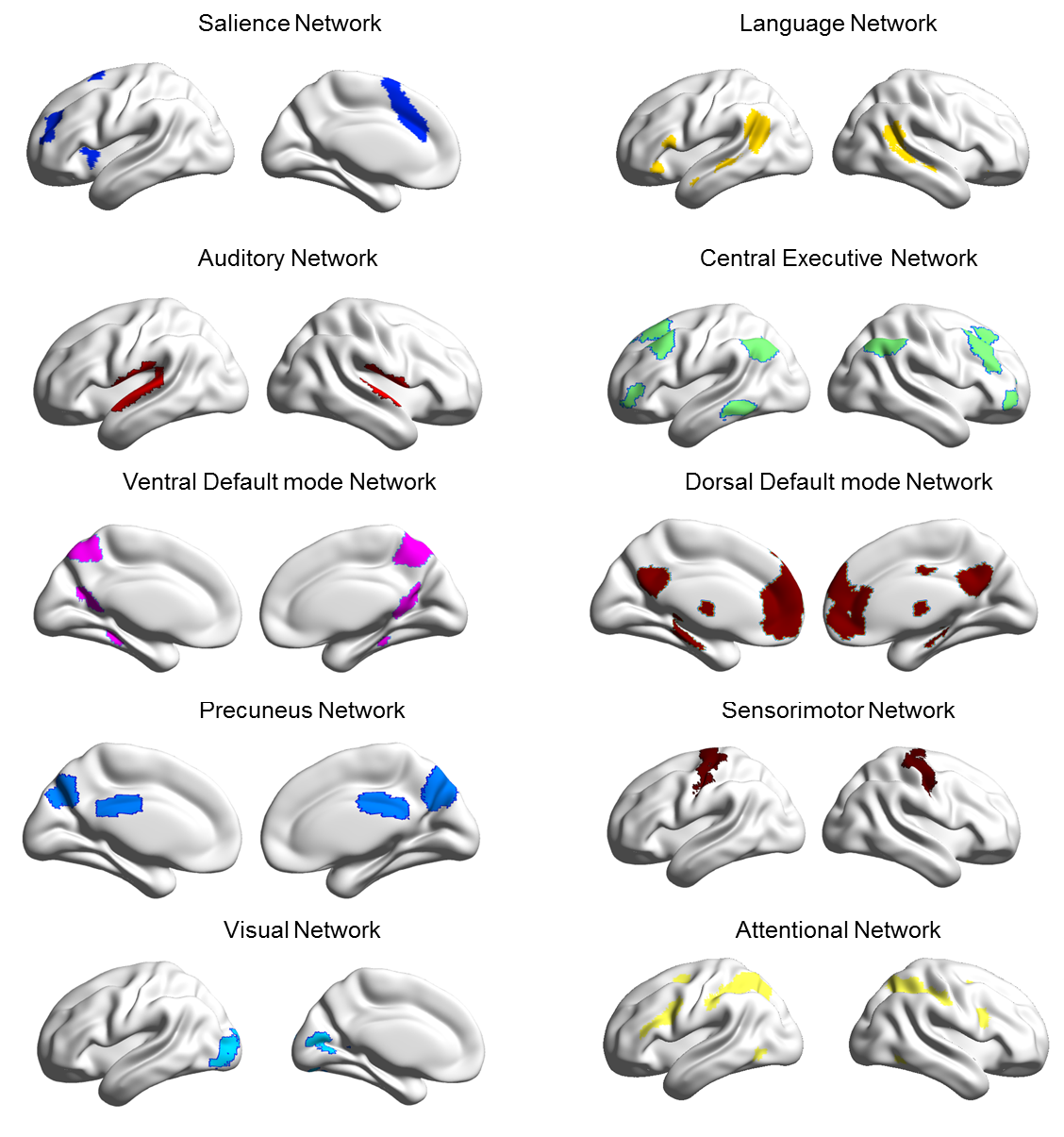


**Supplementary Figure S1. Spatial maps of the 10 resting-state networks (Shirer et al., 2012).**

| **Supplementary Table S1: Regions comprising the resting-state networks as defined in Shirer et al. (2012)** | |
| --- | --- |
| **Resting-State Network** | **Regions** |
| **Dorsal Default Mode Network** | Medial Prefrontal Cortex, Anterior Cingulate Cortex, Orbitofrontal Cortex |
|  | Left Angular Gyrus |
|  | Right Angular Gyrus |
|  | Posterior Cingulate Cortex, Precuneus |
|  | Left and Right Thalamus |
|  | Left Hippocampus |
|  | Right Hippocampus |
|  | Right Superior Frontal Gyrus |
|  | MidCingulate Cortex |
| **Ventral Default Mode Network** | Left Retrosplenial Cortex, Posterior Cingulate Cortex |
|  | Right Retroplenial Cortex, Posterior Cingulate Cortex |
|  | Precuneus |
|  | Left Middle Frontal Cortex |
|  | Right Superior Frontal Gyrus, Middle Frontal Gyrus |
|  | Left Parahippocampal Gyrus |
|  | Right Parahippocampal Gyrus |
|  | Left Middle Occipital Gyrus |
|  | Right Angular Gyrus, Middle Occipital Gyrus |
|  | Right Retroplenial Cortex, Posterior Cingulate Cortex |
|  | Right Lobule IX |
| **Precuneus Network** | MidCingulate Cortex, Posterior Cingulate Cortex |
|  | Precuneus |
|  | Left Angular Gyrus |
|  | Right Angular Gyrus |
| **Salience Network** | Left Insula |
|  | Right Insula |
|  | Left Middle Frontal Gyrus |
|  | Right Middle Frontal Gyrus |
|  | Anterior Cingulate Cortex, Medial Prefrontal Cortex, Supplementary Motor Area |
|  | Left Lobule VI, Crus I |
|  | Right Lobule VI, Crus I |
| **Central Executive Network** | Left Middle Frontal Gyrus, Superior Frontal Gyrus |
|  | Left Inferior Frontal Gyrus, Orbitofrontal Gyrus |
|  | Left Superior Parietal Gyrus, Inferior Parietal Gyrus, Precuneus, Angular Gyrus |
|  | Left Inferior Temporal Gyrus, Middle Temporal Gyrus |
|  | Right Middle Frontal Gyrus, Superior Frontal Gyrus |
|  | Right Middle Frontal Gyrus |
|  | Right Inferior Parietal Gyrus, Supramarginal Gyrus, Angular Gyrus |
|  | Right Crus I |
|  | Left Thalamus |
|  | Left Crus I, Crus II, Lobule VI |
|  | Right Caudate |
| **Language Network** | Inferior Frontal Gyrus |
|  | Left Middle Temporal Gyrus |
|  | Right Inferior Frontal Gyrus |
|  | Left Middle Temporal Gyrus, Angular Gyrus |
|  | Left Middle Temporal Gyrus, Superior Temporal Gyrus, Supramarginal Gyrus, Angular Gyrus |
|  | Right Supramarginal Gyrus, Superior Temporal Gyrus, Middle Temporal Gyrus |
|  | Left Crus I |
| **Sensorimotor Network** | Left Precentral Gyrus, Postcentral Gyrus |
|  | Right Precentral Gyrus, Postcentral Gyrus |
|  | Right Supplementary Motor Area |
|  | Left Thalamus |
|  | Right Thalamus |
|  | Bilateral Lobule IV, Lobule V, Lobule VI |
| **Auditory Network** | Left Superior Temporal Gyrus, Heschl's Gyrus |
|  | Right Superior Temporal Gyrus |
|  | Right Thalamus |
| **Visual Network** | Left Middle Occipital Gyrus, Superior Occipital Gyrus |
|  | Right Middle Occipital Gyrus, Superior Occipital Gyrus |
|  | Calcarine Sulcus |

| **Supplementary Table S2. Definition of non-imaging variables from the Human Connectome Project dataset** | | |
| --- | --- | --- |
| **Category** | **Variable Name** | **Full Description of the Variable** |
| Sleep | PSQI_Latency30Min | Pittsburg Sleep Quality Index: No asleep within 30min |
| Sleep | PSQI_Score | Sleep Quality (Pittsburg Sleep Quality Index: Total Score) |
| Sleep | PSQI_WakeUp^1^ | Pittsburg Sleep Quality Index: Trouble to wake up |
| Sleep | PSQI_AmtSleep | Amount of Sleep (Pittsburg Sleep Quality Index: Total Score) |
| Sleep | PSQI_Min2Asleep^1^ | Pittsburg Sleep Quality Index: Total Score |
| Weight | BMI**^1^** | Body mass Index |
| Drug Use | SSAGA_Mj_Times_Used^1^ | Times used marijuana |
| Drug Use | SSAGA_Mj_Age_1st_Use^1^ | Age at first marijuana use |
| Drug Use | THC^1^ | Use of Marijuana (Test for Marijuana) |
| Alcohol Use | SSAGA_Alc_12_Max_Drinks^1^ | Previous drinking problems (Max drinks in a single day in past 12 months) |
| Alcohol Use | SSAGA_Alc_Hvy_Frq_Drk | Binge drinking (Drinks per day in heaviest 12-month period) (high values indicate low frequency) |
| Smoking Habit | Total_Any_Tobacco_7days^1^ | Tobacco Use (Total times used/smoked any tobacco in past 7 days) |
| Hemoglobin | HbA1C^1^ | Hemoglobin A1C |
| Hematocrit | Hematocrit_1 | Hematocrit Sample 1 |
| Hematocrit | Hematocrit_2 | Hematocrit Sample 2 |
| Blood Pressure | BPSystolic^1^ | Systolic Blood Pressure |
| Blood Pressure | BPDiastolic^1^ | Diastolic Blood Pressure |
| Endurance | Endurance_AgeAdj | Physical Endurance (2-min walk endurance test) |
| Handedness | Handedness | Handedness |
| Family History | FamHist_Moth_None | No Maternal History of psychiatric illness |
| Family History | FamHist_Fath_None | No Paternal History of psychiatric illness |
| Female Only | Menstrual_AgeBegan | age at menarche |
| Female Only | Menstrual_DaysSinceLast | Number of Days since last menstrual cycle ((only used in sex-specific supplemental analysis) |
| Female Only | Menstrual_CycleLength | Length of cycle (only used in sex-specific supplemental analysis) |
| Personality | NEOFAC_C^1^ | NEO-Five-Factor Model Conscientiousness |
| Personality | NEOFAC_E^1^ | NEO-Five-Factor Model Extroversion |
| Personality | NEOFAC_A | NEO-Five-Factor Model Agreeableness |
| Personality | NEOFAC_O | NEO-Five-Factor Model Openness |
| Personality | NEOFAC_N | NEO-Five-Factor Model Neuroticism |
| Life Function | ASR_Totp_T^1^ | Adult self-report Total T score |
| Life Function | ASR_TAO_Sum^1^ | Adult self-report: Sum of Thought, Attention, and Other Problems |
| Life Function | ASR_Intn_T^1^ | Adult self-report internalizing T score |
| Life Function | ASR_Extn_T^1^ | Adult self-report externalizing T score |
| Life Function | ASR_Anxd_Pct^1^ | Adult self-report anxious/depressed |
| Life Function | ASR_Witd_Pct^1^ | Adult self-report withdrawn |
| Life Function | ASR_Soma_Pct^1^ | Adult self-report somatic complains |
| Life Function | ASR_Thot_Pct^1^ | Adult self-report thought problems |
| Life Function | ASR_Attn_Pct^1^ | Adult self-report attention problems |
| Life Function | ASR_Aggr_Pct^1^ | Adult self-report aggressive behavior |
| Life Function | ASR_Rule_Pct^1^ | Adult self-rule breaking behavior |
| Life Function | ASR_Intr_Pct^1^ | Adult self-rule intrusive |
| Emotion | ER40SAD | Penn Emotion Recognition: Correct Sad Identifications |
| Emotion | ER40NOE | Penn Emotion Recognition: Correct Neutral Identifications |
| Emotion | ER40ANG | Penn Emotion Recognition: Correct Anger Identifications |
| Emotion | ER40HAP | Penn Emotion Recognition: Correct Happy Identifications |
| Emotion | ER40FEAR | Penn Emotion Recognition: Correct Fear Identifications |
| Emotion | ER40_CRT^1^ | Penn Emotion Recognition: Correct Responses median Response Time |
| Emotion | ER40_CR | Penn Emotion Recognition: Number of Correct Responses |
| Emotion | Sadness_Unadj | Sadness Survey |
| Emotion | LifeSatisf_Unadj | General Life Satisfaction Survey |
| Emotion | MeanPurp_Unadj | Meaning and Purpose Survey |
| Emotion | PosAffect_Unadj | Positive Affect Survey |
| Emotion | PercReject_Unadj | Perceived Rejection Survey |
| Emotion | EmotSupp_Unadj | Emotional Support Survey |
| Emotion | PercStress_Unadj | Perceived Stress Survey |
| Emotion | SelfEff_Unadj | Self-Efficacy Survey |
| Emotion | AngAffect_Unadj^1^ | Anger-Affect Survey |
| Emotion | AngHostil_Unadj^1^ | Anger Hostility Survey |
| Emotion | AngAggr_Unadj^1^ | Anger-Physical Aggression Survey |
| Emotion | FearAffect_Unadj^1^ | Fear-Affect Survey |
| Emotion | FearSomat_Unadj^1^ | Fear-Somatic Arousal Survey |
| Emotion | Friendship_Unadj | Friendship Survey |
| Emotion | Loneliness_Unadj^1^ | Loneliness Survey |
| Emotion | PercHostil_Unadj^1^ | Perceived hostility Survey |
| Emotion | InstruSupp_Unadj | Instrumental Support Survey |
| Verbal Episodic Memory | IWRD_TOT | Penn Word Memory Test: Number of Correct Responses |
| Verbal Episodic Memory | IWRD_RTC^1^ | Penn Word Memory Test: Correct Responses median Response Time |
| Sustained Attention | SCPT_SEN | Short Penn Continuous Performance Test Sensitivity |
| Sustained Attention | SCPT_SPEC | Short Penn Continuous Performance Test Specificity |
| Sustained Attention | SCPT_TP | Short Penn Continuous Performance Test True Positives |
| Sustained Attention | SCPT_LRNR^1^ | Short Penn Continuous Performance Test Longest Run of Non-Responses |
| Sustained Attention | SCPT_TN | Short Penn Continuous Performance Test True Negatives |
| Sustained Attention | SCPT_FP^1^ | Short Penn Continuous Performance Test False Positives |
| Sustained Attention | SCPT_FN^1^ | Short Penn Continuous Performance Test False Negatives |
| Spatial Orientation | VSPLOT_OFF^1^ | Penn Line Orientation: Total Positions Off for all Trials |
| Spatial Orientation | VSPLOT_CRTE | Penn Line Orientation: Median Reaction Time Divided by expected number of Clicks for correct trials |
| Spatial Orientation | VSPLOT_TC | Penn Line Orientation: Total Number Correct |
| Impulsivity | DDisc_AUC_40K | Delay Discounting: Area under the curve for discounting of $K40 |
| Impulsivity | DDisc_AUC_200 | Delay Discounting: Area under the curve for discounting of $200 |
| Impulsivity | DDisc_SV_1mo_40K | Delay Discounting: Subjective Value for $K40 at 1 month |
| Impulsivity | DDisc_SV_6mo_40K | Delay Discounting: Subjective Value for $K40 at 6 months |
| Impulsivity | DDisc_SV_1yr_40K | Delay Discounting: Subjective Value for $K40 at 1 year |
| Impulsivity | DDisc_SV_3yr_40K | Delay Discounting: Subjective Value for $K40 at 3 years |
| Impulsivity | DDisc_SV_5yr_40K | Delay Discounting: Subjective Value for $K40 at 5 years |
| Impulsivity | DDisc_SV_10yr_40K | Delay Discounting: Subjective Value for $K40 at 10 years |
| Impulsivity | DDisc_SV_1mo_200 | Delay Discounting: Subjective Value for $200 at 1 month |
| Impulsivity | DDisc_SV_6mo_200 | Delay Discounting: Subjective Value for $200 at 6 months |
| Impulsivity | DDisc_SV_1yr_200 | Delay Discounting: Subjective Value for $200 at 1 year |
| Impulsivity | DDisc_SV_3yr_200 | Delay Discounting: Subjective Value for $200 at 3 years |
| Impulsivity | DDisc_SV_5yr_200 | Delay Discounting: Subjective Value for $200 at 5 years |
| Impulsivity | DDisc_SV_10yr_200 | Delay Discounting: Subjective Value for $200 at 10 years |
| Language | PicVocab_AgeAdj | Picture Vocabulary Test |
| Working Memory | ListSort_Unadj | List Sorting Working Memory Test |
| Processing Speed | ProcSpeed_AgeAdj | Pattern Completion Processing Speed scale score |
| Fluid Intelligence | PMAT24_A_SI^1^ | Penn Matrix Test: Total Skipped Items |
| Fluid Intelligence | PMAT24_A_CR | Fluid Intelligence (Penn Matrix Test: Number of Correct Responses) |
| Fluid Intelligence | PMAT24_A_RTCR^1^ | Penn Matrix Test: Median Reaction Time for Correct  Responses |
| Cognitive Status | MMSE_Score | Mini Mental Status Exam Total Score |
| Episodic Memory | PicSeq_AgeAdj | Picture Sequence memory Test |
| Executive Function | CardSort_AgeAdj | Dimensional Change Card Sort Scale Score |
| Executive Function | Flanker_AgeAdj | Flanker Inhibitory Control and Attention Test |
| Language | ReadEng_AgeAdj | Reading Recognition Scale Score |
| Sensory | Mars_Errs^1^ | Errors on Mars Contrast Sensitivity |
| Sensory | Mars_Final | Final Contrast Sensitivity Score |
| Sensory | Mars_Log_Score | Contrast Sensitivity Score |
| Strength | Strength_AgeAdj | Strength test scale score |
| Locomotion | GaitSpeed_Comp | 4-Meter Walk Gait Speed Test |
| Taste | Taste_AgeAdj | Taste Intensity |
| Audition | Noise_Comp | Audition |
| Dexterity | Dexterity_AgeAdj | Pegboard Dexterity test scale score |
| Pain | PainInterf_Tscore | Pain Intensity and Interference |
| Olfaction | Odor_AgeAdj | Odor Identification |
| Education | SSAGA_Educ | Education |
| Date of Acquisition | Acquisition | Quarter of the year during which the data was initially acquired |
| Age | Age_in_Yrs | Age |
| Mean head motion | Mean head movement | Average volume-to-volume head movement |
| More information on the variables: <https://www.humanconnectome.org/documentation/Q3/HCP_Q3_Release_Appendix_VII.pdf>  ^1^ directionality of values reversed in the sparse canonical correlation analysis so that higher values denote better performance, outcome or physical health or health promoting behavior | | |

1. **Properties of resting-state network metastability**

| **Supplementary Table S3. Percentile values of the metastability of each resting-state network** | | | | | |
| --- | --- | --- | --- | --- | --- |
| **Percentile** | **Dorsal Attention Network** | **Language Network** | **Dorsal Default Mode Network** | **Ventral Default Mode Network** | **Precuneus** |
| 1 | 0.1415 | 0.1627 | 0.1602 | 0.1535 | 0.1714 |
| 5 | 0.1488 | 0.1739 | 0.1686 | 0.1640 | 0.1974 |
| 10 | 0.1541 | 0.1780 | 0.1733 | 0.1720 | 0.2099 |
| 15 | 0.1573 | 0.1817 | 0.1777 | 0.1753 | 0.2160 |
| 20 | 0.1596 | 0.1848 | 0.1815 | 0.1776 | 0.2210 |
| 25 | 0.1624 | 0.1873 | 0.1846 | 0.1799 | 0.2235 |
| 30 | 0.1640 | 0.1894 | 0.1865 | 0.1819 | 0.2266 |
| 35 | 0.1657 | 0.1913 | 0.1886 | 0.1843 | 0.2292 |
| 40 | 0.1679 | 0.1934 | 0.1906 | 0.1864 | 0.2313 |
| 45 | 0.1696 | 0.1954 | 0.1925 | 0.1882 | 0.2334 |
| 50 | 0.1712 | 0.1977 | 0.1947 | 0.1904 | 0.2355 |
| 55 | 0.1731 | 0.1997 | 0.1969 | 0.1924 | 0.2370 |
| 60 | 0.1751 | 0.2016 | 0.1984 | 0.1947 | 0.2390 |
| 65 | 0.1771 | 0.2038 | 0.2004 | 0.1965 | 0.2410 |
| 70 | 0.1787 | 0.2059 | 0.2026 | 0.1985 | 0.2428 |
| 75 | 0.1803 | 0.2087 | 0.2051 | 0.2013 | 0.2450 |
| 80 | 0.1823 | 0.2112 | 0.2082 | 0.2040 | 0.2474 |
| 85 | 0.1853 | 0.2153 | 0.2112 | 0.2065 | 0.2495 |
| 90 | 0.1892 | 0.2193 | 0.2163 | 0.2104 | 0.2525 |
| 95 | 0.1948 | 0.2255 | 0.2230 | 0.2170 | 0.2572 |
| 99 | 0.2033 | 0.2366 | 0.2329 | 0.2284 | 0.2645 |
| **Percentile** | **Central Executive Network** | **Visual Network** | **Salience Network** | **Sensorimotor Network** | **Auditory Network** |
| 1 | 0.1514 | 0.1720 | 0.1709 | 0.1716 | 0.1996 |
| 5 | 0.1606 | 0.1964 | 0.1816 | 0.1836 | 0.2205 |
| 10 | 0.1667 | 0.2053 | 0.1875 | 0.1902 | 0.2286 |
| 15 | 0.1712 | 0.2100 | 0.1908 | 0.1939 | 0.2329 |
| 20 | 0.1749 | 0.2147 | 0.1934 | 0.1968 | 0.2355 |
| 25 | 0.1776 | 0.2177 | 0.1953 | 0.1991 | 0.2375 |
| 30 | 0.1800 | 0.2202 | 0.1984 | 0.2008 | 0.2393 |
| 35 | 0.1825 | 0.2221 | 0.2006 | 0.2028 | 0.2407 |
| 40 | 0.1845 | 0.2242 | 0.2020 | 0.2047 | 0.2423 |
| 45 | 0.1860 | 0.2260 | 0.2037 | 0.2069 | 0.2439 |
| 50 | 0.1875 | 0.2283 | 0.2052 | 0.2084 | 0.2452 |
| 55 | 0.1900 | 0.2307 | 0.2074 | 0.2101 | 0.2463 |
| 60 | 0.1920 | 0.2324 | 0.2091 | 0.2119 | 0.2478 |
| 65 | 0.1941 | 0.2340 | 0.2111 | 0.2139 | 0.2491 |
| 70 | 0.1956 | 0.2362 | 0.2129 | 0.2156 | 0.2505 |
| 75 | 0.1975 | 0.2383 | 0.2150 | 0.2183 | 0.2522 |
| 80 | 0.2005 | 0.2406 | 0.2170 | 0.2207 | 0.2542 |
| 85 | 0.2030 | 0.2428 | 0.2199 | 0.2234 | 0.2563 |
| 90 | 0.2076 | 0.2464 | 0.2237 | 0.2275 | 0.2590 |
| 95 | 0.2137 | 0.2519 | 0.2294 | 0.2330 | 0.2625 |
| 99 | 0.2262 | 0.2600 | 0.2390 | 0.2423 | 0.2685 |

**Supplementary Figure S2. Percentile curves of the metastability of each resting-state network.**

PN: precuneus network; AN: auditory network; VN: visual network; SMN: sensorimotor network; SAL: salience network; vDMN: ventral default mode network; CEN: central executive network; dDMN: dorsal default mode network; LN: language network; DAN: dorsal attention network.

**Supplementary Figure S3. Histograms of the metastability of each resting-state network**

The red line represents the fitted normal distribution curve. PN: precuneus network; AN: auditory network; VN: visual network; SMN: sensorimotor network; SAL: salience network; vDMN: ventral default mode network; CEN: central executive network; dDMN: dorsal default mode network; LN: language network; DAN: dorsal attention network.

| **Supplementary Table S4. Bonferroni corrected pairwise comparisons of the metastability of the resting-state networks** | | | |
| --- | --- | --- | --- |
| **Pairs of networks considered** | **Paired t-test derived mean difference in metastability** | **95% Confidence Intervals**  **(lower, upper bound)** | **p-value** |
| AN - PN | 0.011 | 0.009, 0.014 | < 1e-15 |
| AN - VN | 0.017 | 0.015, 0.020 | < 1e-15 |
| AN - SMN | 0.036 | 0.033, 0.038 | < 1e-15 |
| AN - SAL | 0.038 | 0.036, 0.041 | < 1e-15 |
| AN - LN | 0.046 | 0.043, 0.048 | < 1e-15 |
| AN - dDMN | 0.049 | 0.047,0.052 | < 1e-15 |
| AN - vDMN | 0.053 | 0.051, 0.056 | < 1e-15 |
| AN - CEN | 0.056 | 0.054, 0.059 | < 1e-15 |
| AN - DAN | 0.072 | 0.070, 0.075 | < 1e-15 |
| PN - VN | 0.006 | 0.004, 0.009 | 8.49e-14 |
| PN - SMN | 0.024 | 0.022, 0.027 | < 1e-15 |
| PN - SAL | 0.027 | 0.025, 0.030 | < 1e-15 |
| PN - LN | 0.035 | 0.032, 0.037 | < 1e-15 |
| PN - dDMN | 0.038 | 0.035, 0.040 | < 1e-15 |
| PN - vDMN | 0.042 | 0.040, 0.045 | < 1e-15 |
| PN - CEN | 0.045 | 0.043, 0.048 | < 1e-15 |
| PN - DAN | 0.061 | 0.059, 0.064 | < 1e-15 |
| VN - SMN | 0.018 | 0.016, 0.021 | < 1e-15 |
| VN - SAL | 0.021 | 0.019, 0.024 | < 1e-15 |
| VN - LN | 0.029 | 0.026, 0.031 | < 1e-15 |
| VN - dDMN | 0.032 | 0.029, 0.034 | < 1e-15 |
| VN - vDMN | 0.036 | 0.034, 0.039 | < 1e-15 |
| VN - CEN | 0.039 | 0.037, 0.042 | < 1e-15 |
| VN - DAN | 0.055 | 0.053, 0.058 | < 1e-15 |
| SMN - SAL | 0.003 | 0.000, 0.005 | 0.005 |
| SMN - LN | 0.010 | 0.008, 0.013 | < 1e-15 |
| SMN - dDMN | 0.014 | 0.011, 0.016 | < 1e-15 |
| SMN - vDMN | 0.018 | 0.015, 0.020 | < 1e-15 |
| SMN - CEN | 0.021 | 0.018, 0.023 | < 1e-15 |
| SMN - DAN | 0.037 | 0.034, 0.039 | < 1e-15 |
| SAL - LN | 0.007 | 0.005, 0.010 | < 1e-15 |
| SAL - dDMN | 0.011 | 0.008, 0.013 | < 1e-15 |
| SAL - vDMN | 0.015 | 0.012, 0.017 | < 1e-15 |
| SAL - CEN | 0.018 | 0.015, 0.020 | < 1e-15 |
| SAL - DAN | 0.034 | 0.032, 0.037 | < 1e-15 |
| LN - dDMN | 0.003 | 0.001, 0.006 | 0.0006 |
| LN - vDMN | 0.008 | 0.005, 0.010 | < 1e-15 |
| LN - CEN | 0.011 | 0.008, 0.013 | < 1e-15 |
| LN - DAN | 0.027 | 0.024, 0.029 | < 1e-15 |
| dDMN - vDMN | 0.004 | 0.002, 0.007 | 1.3e-06 |
| dDMN - CEN | 0.007 | 0.005, 0.010 | < 1e-15 |
| dDMN - DAN | 0.023 | 0.021, 0.026 | < 1e-15 |
| vDMN - CEN | 0.003 | 0.000, 0.005 | 0.005 |
| vDMN - DAN | 0.019 | 0.017, 0.022 | < 1e-15 |
| CEN - DAN | 0.016 | 0.014, 0.019 | < 1e-15 |
| Bonferroni adjusted value p=0.001 (0.05/45 pairwise comparisons); Networks: AN: Auditory; DMN: Default mode; PN: Precuneus; VN: Visual; SMN: Sensorimotor; SAL: Salience; LN: Language; dDMN: dorsal DMN; vDMN: ventral DMN; CEN: Central Executive; DAN: Dorsal Attention | | | |


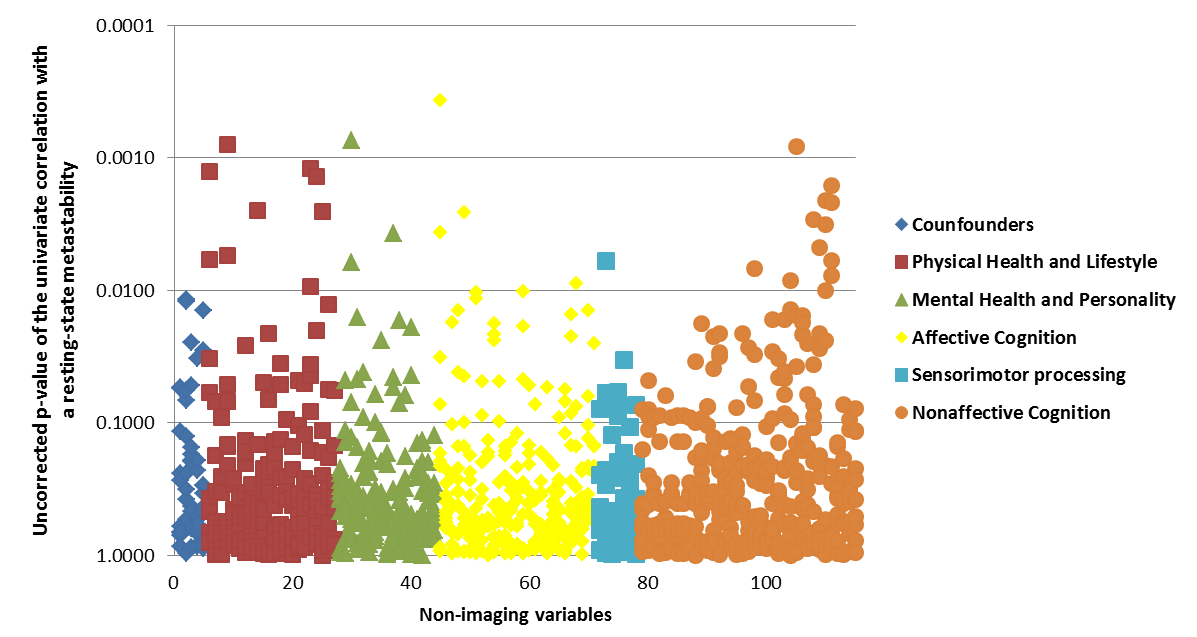


**Supplementary Figure S4. Univariate Pearson’s correlations between the variables of the resting-state metastability dataset and the non-imaging dataset**. None were significant following FDR-correction.

| **Supplementary Table S5. Canonical correlation coefficients and weights between the metastability variate and the non-imaging variables** | | |
| --- | --- | --- |
| Variable | Full dataset | Weight in full dataset |
|  | r |  |
| PMAT24_A_CR | 0.15 | 0.39 |
| PicVocab_AgeAdj | 0.13 | 0.33 |
| ReadEng_AgeAdj | 0.13 | 0.32 |
| Endurance_AgeAdj | 0.12 | 0.30 |
| ListSort_Unadj | 0.12 | 0.28 |
| ER40SAD | 0.11 | 0.24 |
| PMAT24_A_RTCR | 0.08 | - |
| Flanker_AgeAdj | 0.08 | 0.14 |
| ProcSpeed_AgeAdj | 0.07 | 0.12 |
| VSPLOT_TC | 0.07 | 0.12 |
| PSQI_AmtSleep | 0.07 | 0.11 |
| InstruSupp_Unadj | 0.06 | 0.09 |
| GaitSpeed_Comp | 0.06 | 0.07 |
| PicSeq_AgeAdj | 0.05 | 0.06 |
| MMSE_Score | 0.05 | 0.05 |
| SSAGA_Alc_12_Max_Drinks | 0.05 | - |
| Handedness | 0.05 | 0.05 |
| DDisc_SV_10yr_40K | 0.05 | 0.04 |
| FearAffect_Unadj | 0.05 | - |
| PercHostil_Unadj | 0.04 | - |
| Menstrual_AgeBegan | 0.04 | 0.02 |
| ASR_Anxd_Pct | 0.04 | - |
| NEOFAC_O | 0.04 | 0.01 |
| Loneliness_Unadj | 0.04 | - |
| LifeSatisf_Unadj | 0.04 | - |
| DDisc_SV_1yr_200 | 0.04 | - |
| NEOFAC_A | 0.03 | - |
| ER40_CR | 0.03 | - |
| CardSort_AgeAdj | 0.03 | - |
| Total_Any_Tobacco_7days | 0.03 | - |
| DDisc_AUC_40K | 0.03 | - |
| DDisc_SV_3yr_200 | 0.03 | - |
| DDisc_AUC_200 | 0.03 | - |
| PercStress_Unadj | 0.03 | - |
| DDisc_SV_5yr_40K | 0.03 | - |
| Dexterity_AgeAdj | 0.02 | - |
| DDisc_SV_10yr_200 | 0.02 | - |
| DDisc_SV_3yr_40K | 0.02 | - |
| DDisc_SV_6mo_200 | 0.02 | - |
| FearSomat_Unadj | 0.02 | - |
| DDisc_SV_5yr_200 | 0.02 | - |
| ASR_Intn_T | 0.02 | - |
| SCPT_LRNR | 0.02 | - |
| SCPT_TN | 0.02 | - |
| SCPT_SPEC | 0.02 | - |
| PainInterf_Tscore | 0.02 | - |
| ASR_Soma_Pct | 0.02 | - |
| NEOFAC_N | 0.02 | - |
| ASR_Attn_Pct | 0.02 | - |
| AngHostil_Unadj | 0.01 | - |
| Taste_AgeAdj | 0.01 | - |
| AngAffect_Unadj | 0.01 | - |
| DDisc_SV_1yr_40K | 0.01 | - |
| IWRD_TOT | 0.01 | - |
| ASR_TAO_Sum | 0.00 | - |
| Mars_Final | 0.00 | - |
| ER40NOE | 0.00 | - |
| PercReject_Unadj | 0.00 | - |
| Friendship_Unadj | 0.00 | - |
| Sadness_Unadj | 0.00 | - |
| ER40HAP | 0.00 | - |
| Odor_AgeAdj | 0.00 | - |
| SCPT_SEN | 0.00 | - |
| SCPT_TP | 0.00 | - |
| ASR_Witd_Pct | 0.00 | - |
| Mars_Log_Score | 0.00 | - |
| SCPT_FN | 0.00 | - |
| EmotSupp_Unadj | 0.00 | - |
| ER40ANG | 0.00 | - |
| PosAffect_Unadj | 0.00 | - |
| DDisc_SV_1mo_200 | 0.00 | - |
| Mars_Errs | -0.01 | - |
| ASR_Totp_T | -0.01 | - |
| SSAGA_Mj_Age_1st_Use | -0.01 | - |
| DDisc_SV_1mo_40K | -0.01 | - |
| ASR_Thot_Pct | -0.01 | - |
| Menstrual_DaysSinceLast | -0.01 | - |
| PSQI_Latency30Min | -0.02 | - |
| SCPT_FP | -0.02 | - |
| Strength_AgeAdj | -0.02 | - |
| AngAggr_Unadj | -0.02 | - |
| SSAGA_Mj_Times_Used | -0.02 | - |
| SelfEff_Unadj | -0.02 | - |
| PSQI_WakeUp | -0.02 | - |
| Hematocrit_2 | -0.02 | **-** |
| MeanPurp_Unadj | -0.02 | **-** |
| Hematocrit_1 | -0.03 | **-** |
| PSQI_Score | -0.03 | **-** |
| ASR_Intr_Pct | -0.03 | - |
| BPDiastolic | -0.03 | - |
| ASR_Aggr_Pct | -0.03 | - |
| VSPLOT_CRTE | -0.04 | - |
| Noise_Comp | -0.04 | - |
| ER40FEAR | -0.04 | - |
| DDisc_SV_6mo_40K | -0.04 | - |
| Menstrual_CycleLength | -0.04 | - |
| ER40_CRT | -0.04 | -0.01 |
| ASR_Rule_Pct | -0.04 | -0.02 |
| PSQI_Min2Asleep | -0.04 | -0.02 |
| THC | -0.04 | -0.02 |
| ASR_Extn_T | -0.05 | -0.03 |
| VSPLOT_OFF | -0.05 | -0.06 |
| IWRD_RTC | -0.06 | -0.07 |
| NEOFAC_E | -0.06 | -0.08 |
| HbA1C | -0.06 | -0.08 |
| BPSystolic | -0.06 | -0.10 |
| SSAGA_Alc_Hvy_Frq_Drk | -0.07 | -0.11 |
| NEOFAC_C | -0.10 | -0.22 |
| BMI | -0.12 | -0.28 |
| PMAT24_A_SI | -0.14 | -0.37 |


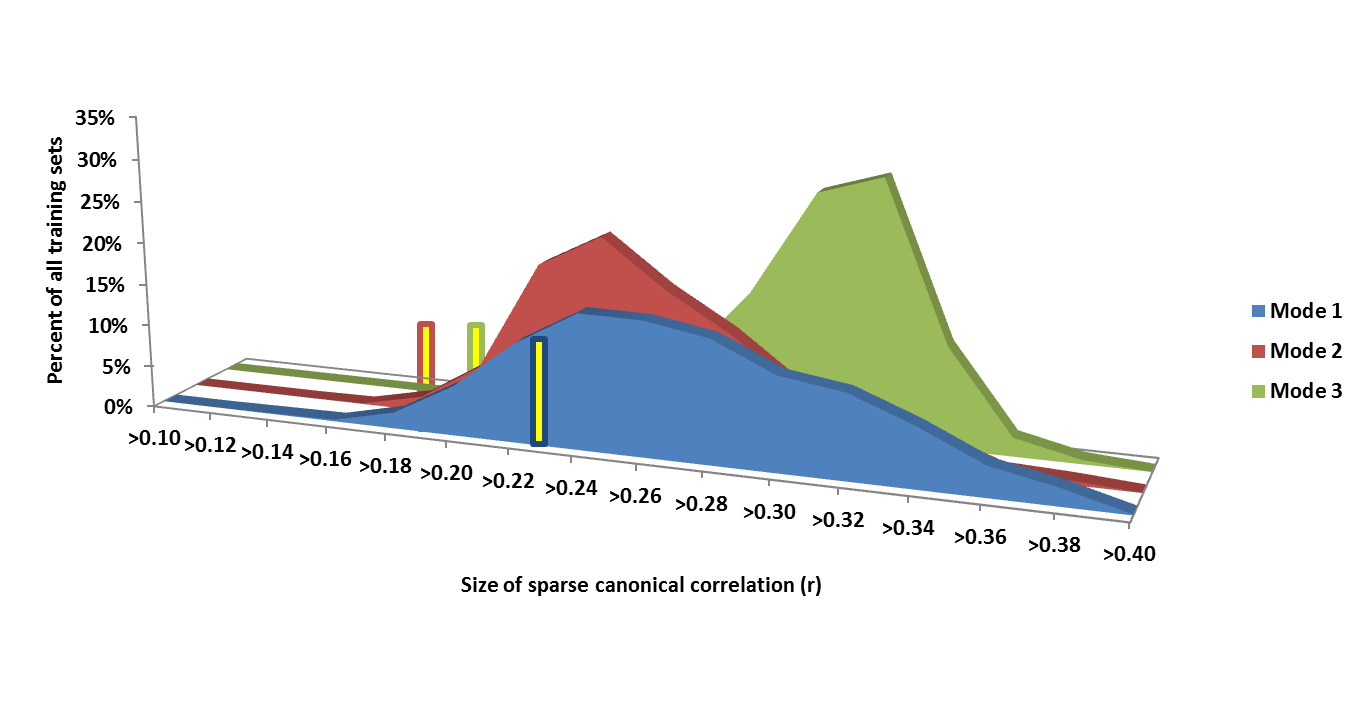


**Supplementary Figure S5.** Distribution of the sparse canonical correlation coefficients based on 10,000 randomly resampled subsets of half the sample (n=409). Yellow bars indicate the sCCA r-value of the model based on the original sample (n=818). Modes indicate component pairs (e.g., Mode 1=first component pair). Mode 1 was significant (see main text) and showed minimal overfitting as indicated in this figure. Modes 2 and 3 were not statistically significant (see main text) and were also not reliable as shown here.

| **Supplementary Table S6. Canonical correlation coefficients and weights between the non-imaging variate and the metastability variables** | | |
| --- | --- | --- |
| Variable | Full dataset | Weight in full dataset |
|  | r |  |
| Ventral Default Mode Network | 0.16 | 0.58 |
| Central Executive Network | 0.14 | 0.47 |
| Dorsal Attention Network | 0.12 | 0.37 |
| Dorsal Default Mode Network | 0.12 | 0.36 |
| Precuneus (part of the Default Mode Network) | 0.10 | 0.28 |
| Auditory Network | 0.09 | 0.26 |
| Sensorimotor Network | 0.07 | 0.15 |
| Language Network | 0.02 | - |
| Visual Network | 0.03 | - |
| Salience Network | 0.00 | - |

**
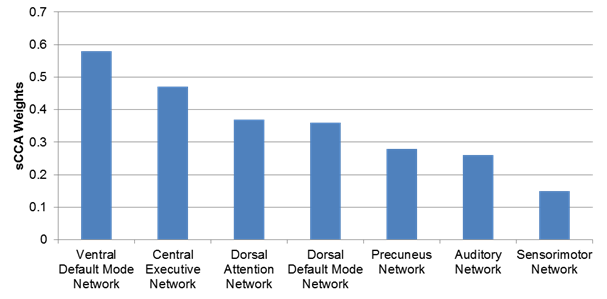
**

**Supplementary Figure S6. Sparse Canonical Correlation Analysis (sCCA) weights of the resting-state networks with the non-imaging variate (further details in Supplemental Table S6)**

**Supplemental sCCA**

We conducted a further sCCA analysis following the same procedures described in the main text; this supplemental sCCA involved the non-imaging dataset and a new dataset that comprised the pairwise differences in the RSN metastability. This sCCA was not significant (r=0.18, p=0.48) and suggests that it is primarily the metastability of each RSN that is associated with human traits rather than the degree of pairwise difference in metastability between networks.

**C. Potential Confounders**

**Confounders in the sCCA model (age, education, head motion and date of acquisition)**

Supplemental Table S7 shows the weights of the confounders entered in the sCCA model. The weights of these variables were minimal and did not influence the model. This is further supported by the univariate analyses. Specifically, the univariate associations between resting state metastability and average head motion are shown in Supplementary Table S8. Univariate general linear models with “date of acquisition” as a fixed factor and the metastability of each of the RSNs as the dependent variable was statistically significant for two networks: CEN: F=2.34, p=0.008; dDMN: F=2.10, p=0.018 (uncorrected).

**Sex**

Univariate general linear models with sex as a fixed factor and the metastability of each of the RSNs as the dependent variable were not statistically significant (all F<3.8, all p>0.06). We also repeated the sCCA including sex as a variable in the non-imaging dataset. The weight of sex in the model was 0 and the overall association between the two datasets was unchanged (r-value of the sCCA including sex was r =0.23).

| **Supplementary Table S7. Association of the metastability variate with *potentially confounding variables* in the non-imaging dataset (all values refer to the full dataset)** | | |
| --- | --- | --- |
| Variable | r-value | Weight |
| Education | 0.04 | - |
| Date of Acquisition | 0.03 | - |
| Age | -0.02 | - |
| Mean head motion | -0.08 | -0.16 |

| **Supplementary Table S8. Univariate correlations between resting-state metastability and head motion** | | |
| --- | --- | --- |
| Network | r-value | p-value (uncorrected) |
| Salience Network | 0.027 | 0.43 |
| Dorsal Default Mode Network | 0.005 | 0.88 |
| Sensorimotor Network | 0.004 | 0.91 |
| Dorsal Attention Network | 0.001 | 0.99 |
| Language Network | -0.010 | 0.76 |
| Visual Network | -0.018 | 0.60 |
| Auditory Network | -0.031 | 0.37 |
| Precuneus | -0.032 | 0.36 |
| Ventral Default Mode Network | -0.073 | 0.04 |
| Central Executive Network | -0.083 | 0.02 |

1. **Network Synchrony**

We estimated the synchrony of each RSN, defined as the mean of the Kuramoto order parameter over time, as described in the main text. Supplemental Figure S7 shows the distribution of synchrony for each resting-state network (RSN) across the 818 participants of the Human Connectome Project. The univariate Pearson’s correlation coefficients between RSNs’ metastability and synchrony are shown in Supplementary Table S9. We conducted a sCCA between the synchrony dataset and the non-imaging dataset using the same methodology as for metastability described in the main text. The association between the synchrony and non-imaging datasets was modest and significant (r=0.246, p=0.002). The non-imaging variables with the highest weight represented physical traits relating to perceptual contrast sensitivity (weight =-0.41), amount of sleep (weight=-0.29) and manual dexterity (weight=-0.24) and substance use relating to cannabis (weight=0.40) and maximum of alcoholic drinks in a single day within the last year (weight=0.23). The networks whose synchrony was associated with the non-imaging variate included the vDMN (weight=0.64), the dDMN (weight=0.55), the DAN (weight=0.50) and the CEN (weight=0.16).


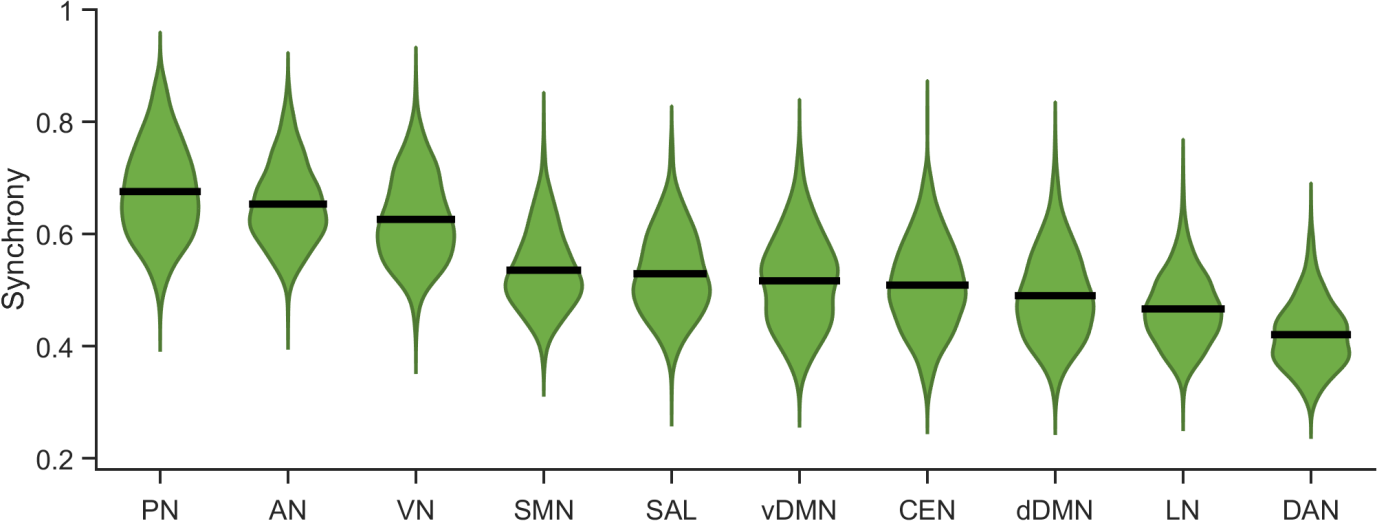


**Supplementary Figure S7. Violin plots of the distribution of synchrony for each resting-state network (RSN) across the 818 participants of the Human Connectome Project.** The solid black lines depict mean values across participants. PN: precuneus network; AN: auditory network; VN: visual network; SMN: sensorimotor network; SAL: salience network; vDMN: ventral default mode network; CEN: central executive network; dDMN: dorsal default mode network; LN: language network; DAN: dorsal attention network.

| **Supplementary Table S9. Univariate correlations between metastability and synchrony for each resting-state network**. | | |
| --- | --- | --- |
| Network | r-value | p-value |
| Precuneus Network | -0.62 | 3e-87 |
| Auditory Network | -0.40 | 4e-33 |
| Visual Network | -0.10 | 0.0038 |
| Sensorimotor Network | 0.31 | 3e-20 |
| Salience Network | 0.32 | 5e-21 |
| Ventral Default Mode Network | 0.20 | 9e-09 |
| Central Executive Network | 0.35 | 2e-25 |
| Dorsal Default Mode Network | 0.56 | 4e-68 |
| Language Network | 0.59 | 2e-78 |
| Dorsal Attention Network | 0.52 | 2e-58 |
